# Recent Advances in Polymers as Matrices for Drug Delivery Applications

**DOI:** 10.3390/ph16121674

**Published:** 2023-12-01

**Authors:** Zoilo González, Ana Ferrandez-Montero, Juan Domínguez-Robles

**Affiliations:** 1 BioPrEn Group (RNM940), Chemical Engineering Department, Instituto Químico para la Energía y el Medioambiente (IQUEMA), Faculty of Science, Universidad de Córdoba (UCO), 14014 Córdoba, Spain; 2 Instituto de Cerámica y Vidrio, Consejo Superior de Investigaciones Científicas (CSIC), Campus de Cantoblanco, c/Kelsen 5, 28049 Madrid, Spain; 3 Department of Pharmacy and Pharmaceutical Technology, Faculty of Pharmacy, Universidad de Sevilla, 41012 Seville, Spain

Polymeric-based drug delivery systems have become versatile and valuable candidates in sectors such as pharmaceuticals, health, medicine, etc., due to a unique set of characteristics that makes them well-suited for many specific applications. Their tunable physicochemical properties, biocompatibility, and capability to encapsulate and release a wide variety of therapeutic agents are just a few examples of their extensive potential [1].

The targeted delivery of a drug towards an organ, a tissue, or unhealthy cells by carriers is currently one of the major challenges facing our scientific community. In recent years, there has been a surge in research driven by the necessity for improved therapeutic efficacy, reduced side effects, and enhanced patient compliance, among other factors. In this sense, advancements in polymer science have paved the way for the design of sophisticated delivery platforms with controlled release kinetics, targeting capabilities, and improved stability for encapsulated drugs. Various examples of drug delivery devices exist, such as orodispersible tablets and films, solid oral dosage forms, drug-eluting stents, therapeutic contact lenses, subcutaneous implants designed for prolonged drug delivery, and microneedles for transdermal drug delivery [2].

In recent years, researchers have paid increasing attention to the design, processing, and controlled use of advanced polymeric-based formulations [3,4]. Such aspects of polymer structures play an important role in current drug delivery, since the demands on delivery vehicles are increasing as regards drug release rate, drug solubilisation capacity, the minimisation of drug degradation, the reduction of drug toxicity, etc. [5].

Taking all the above-mentioned into account, the articles and review included in this editorial aim to address some of the most recent advances and challenges that have arisen around the topic. The scientific community plays a very important role in generating the basic know-how that gives rise to pharmaceutical technology and allows developments in the laboratory to be transferred to society. Some of the most cutting-edge developments, challenges, and future prospects involving these topics, have been reported in this Special Issue.

Abdelkader et al. introduced smartFilm technology, where the drug is embedded within a cellulose-based paper matrix in an amorphous state, to enhance the solubility of orally administered drugs (Contribution I). They embedded drugs in cellulose-based paper, improving solubility, but faced challenges with the paper’s flowability and compression behaviour. Their study converted paper into granules using wet granulation and tested different sucrose binders. Granules with ≤30% sucrose content produced tablets meeting pharmaceutical standards. This approach offers the potential for the large-scale production of paper-based tablets.

László et al. aimed to develop an innovative transdermal therapy system for chronic pain using a slow hydrogen sulphide (H_2_S) donor, diallyl disulphide (DADS), as a model compound (Contribution II). They found that DADS had optimal solubility in dimethyl silicone oil, and the rate of diffusion correlated with the oil content in the patch. The 8-day-old patch provided better-controlled drug release compared to the 4-day-old one. This study established a promising silicone-based matrix for the stable storage and controlled release of DADS, providing a suitable transdermal therapeutic system for chronic pain treatment.

In the next study, Heczko et al. explored the thermal properties, molecular dynamics, crystallisation kinetics, and intermolecular interactions of binary mixtures (BMs) containing flutamide (FL) and various poly(N-vinylpyrrolidone) (PVP) polymers, including those synthesised under high pressure, which differ in their microstructure (Contribution III). They found significant differences in glass transition temperature (T*_g_*) between FL and synthetised PVP mixtures with different excipient levels (10 and 30 wt%). This effect was less pronounced in the FL and commercial PVP system. Therefore, this unexpected finding in the former mixtures was linked to polymer microstructure variations. Additionally, the onset temperature of FL crystallisation was suppressed in BMs composed of synthesised polymers. Thus, this research highlighted the influence of the polymer microstructure on pharmaceutical physical stability, an overlooked issue in the research literature.

Park et al. performed a study to investigate the potential of gene therapy as an alternative to chemotherapy (Contribution IV). For this purpose, the authors manufactured gene carrier nanoparticles from a biocompatible and biodegradable polymer, L-tyrosine polyurethane (LTU). These nanoparticles were fabricated from presynthesised desaminotyrosyl tyrosine hexyl ester (DTH) and polyethylene glycol (PEG), through double emulsion and solvent evaporation techniques, resulting in porous nanoparticles. These nanoparticles were effective in delivering genes and drugs and demonstrated excellent cell adsorption abilities. Overall, these findings suggested that LTU nanoparticles had great potential as gene carriers for effective gene therapy.

Small-diameter synthetic vascular grafts are essential for surgical bypass procedures, particularly when suitable autologous vessels are unavailable due to factors like prior surgeries [6,7]. Thrombosis is the leading cause of failure of these grafts when used for this revascularisation technique [8,9,10]. Therefore, the development of biocompatible and biodegradable vascular grafts capable of providing a localised and sustained release of antithrombotic drugs represents a crucial advancement in combating cardiovascular diseases [11,12,13,14]. Accordingly, the study developed by Domínguez-Robles et al. and published in this Special Issue focused on developing biocompatible and biodegradable antiplatelet tubular grafts for cardiovascular use through semi-solid extrusion (SSE) 3D printing technology (Contribution V). The grafts were able to release a model antithrombotic drug, acetylsalicylic acid (ASA), for up to 2 weeks. As expected, grafts with 10% (*w*/*w*) ASA exhibited lower platelet adhesion than blank grafts or those with 5% (*w*/*w*) ASA. Additionally, grafts containing 1% rifampicin (RIF) inhibited the growth of *Staphylococcus aureus*. Importantly, the incorporation of ASA and RIF into the graft composition did not compromise cell viability or proliferation during short incubation periods.

The same authors also highlighted the great potential of different 3D printing techniques, including fused deposition modelling and SSE for the manufacture of small-diameter synthetic vascular grafts from drug-loaded biocompatible and biodegradable polymeric matrices [15]. Three-dimensional printing technology holds a distinct advantage over alternative techniques due to its capacity to efficiently produce devices of multiple shapes and sizes [16,17]. This feature not only simplifies the manufacturing process, but also accelerates it significantly. Moreover, it underscores the immense potential of 3D printing techniques in the development and production of medical devices [18,19], including cardiovascular grafts [20].

Lastly, in the only review published in this Special Issue (Contribution VI), the authors explored transdermal drug delivery methods, highlighting the emerging use of microneedles (MNs) as minimally invasive devices which are capable of bypassing the skin barrier to provide both systemic and localised pharmacological effects. Carbohydrates are key materials in MN production due to their versatile properties. The review covered specific carbohydrates, drug delivery strategies, and mechanical properties of carbohydrate-based MNs. It also examined progress in clinical translation and the potential for commercialisation. Therefore, this review summarised the future prospects of carbohydrate-based MNs, positioning them as a novel category of topical drug delivery systems.
